# Rapidly progressive polyneuropathy due to dry beriberi in a man: a case report

**DOI:** 10.1186/1752-1947-4-409

**Published:** 2010-12-21

**Authors:** Anthony J Howard, Omesh Kulkarni, Godwin Lekwuwa, Hedley CA Emsley

**Affiliations:** 1 Department of Neurology, Royal Preston Hospital, Sharoe Green Lane, Preston, PR2 9HT, UK; 2 Department of Neurophysiology, Royal Preston Hospital, Sharoe Green Lane, Preston, PR2 9HT, UK

## Abstract

**Introduction:**

We describe a case of rapidly progressive and severely debilitating polyneuropathy in a patient with confirmed hypovitaminosis B1, consistent with dry beriberi. Crucially, this is a treatable condition, although sometimes with incomplete recovery, but it is probably under-recognized yet increasingly common given increasing levels of alcohol abuse in the western world.

**Case presentation:**

A 49-year-old Caucasian British man presented with progressive weakness of both lower limbs of approximately seven months' duration. He noted difficulty climbing stairs. He also complained of lethargy, and loss of muscle bulk, including his thighs. He had a history of type 2 diabetes mellitus and admitted prior alcohol abuse but denied excessive alcohol intake in the five years prior to presentation. Initial clinical and neurophysiological examinations were consistent with a mild peripheral neuropathy and probable proximal myopathy. However, over the subsequent four months he evolved a marked tetraparesis, with profound sensory disturbance of all limbs. Repeat neurophysiology revealed a widespread polyneuropathy with extensive acute and sub-acute denervation changes in all four limbs, and reduced or absent sensory nerve action potentials. Hypovitaminosis B1 was confirmed (45 nmol/L, reference range 66-200 nmol/L). His rapid clinical deterioration was in keeping with dry beriberi. He was treated with thiamine. Subsequent follow-up revealed slow but significant improvement, such that by 15-16 months from the initial onset of symptoms, and approximately six months after the onset of his marked tetraparesis, he was able to stand independently and was gradually gaining confidence in walking pending a period of in-patient neurorehabilitation.

**Conclusion:**

A potentially wide differential diagnosis exists for this type of presentation. Confirming hypovitaminosis B1 by requesting the assay prior to vitamin replacement ensures accurate diagnosis and appropriate ongoing treatment. An increasingly high index of suspicion is likely to be required in the context of increasing levels of alcohol abuse in the western world and the possible increasing prevalence of dry beriberi.

## Introduction

Dry beriberi or 'acute nutritional polyneuropathy' is considered to be rare in the western world. Rapid deterioration can occur, typically with weakness, paraesthesiae and neuropathic pain. Striking motor nerve involvement can occur, mimicking Guillain-Barré syndrome (GBS). Indeed, a GBS-like deterioration has previously been reported [1,2]. In the context of increasing alcohol abuse in the western world, it is possible that alcoholic neuropathy associated with abrupt deterioration due to concomitant nutritional hypovitaminosis B1 may be seen increasingly often.

### Case presentation

#### Initial presentation

A 49-year-old Caucasian British man with a long history of type 2 diabetes mellitus (DM) and excessive alcohol intake presented with progressive weakness of both lower limbs of approximately seven months' duration. He was morbidly obese with a body mass index (BMI) of 45. Over the same period he had noticed thinning of his muscles, particularly noticeable in his thighs. As well as lethargy, he had recently gained weight. Previously he had consumed very high levels of alcohol, but he did not admit to excessive alcohol intake in the five years prior to this presentation. He remained ambulant. Abnormalities on clinical examination included grade 4 power throughout both his lower limbs, absent ankle jerks and glove and stocking distribution reduction in pinprick sensation to his mid forearms and proximal thighs. Initial electromyogram (EMG)/nerve conduction studies (NCS) demonstrated a mild sensory peripheral polyneuropathy (consistent with long standing type 2 DM and chronic alcohol abuse). There were also patchy non-specific EMG abnormalities suggesting a non-specific myopathic process.

#### Clinical course

Rapid deterioration occurred over the course of approximately three months, with relatively rapid evolution of tetraparesis. Urgent admission was arranged, by which stage he was unable to walk. He had also developed tingling in his legs and arms. On examination, his cranial nerves remained intact but he had marked weakness of upper limbs (grade 3) and lower limbs (grade 2), with absent lower limb and reduced upper limb deep tendon reflexes bilaterally. He also had prominent sensory impairment in his upper and lower limbs, including loss of pain sensation to his waist. At one stage this was suggestive of a possible spinal sensory level. Repeat EMG/NCS four months after the first study showed evidence of a widespread polyneuropathy, with sensory nerve action potentials absent or reduced and evidence of extensive acute and sub-acute denervation in his upper and lower limbs. Although an EMG showed extensive motor denervation suggestive of anterior horn cell disease, this could be ruled out due to the extensive degree of sensory impairment.

#### Other investigations

His *c*erebrospinal fluid was acellular with normal protein and glucose concentrations. Oligoclonal bands were absent. A muscle biopsy of his left quadriceps showed mild non-specific morphological abnormalities with mainly selective type 2-fibre atrophy, possibly associated with an element of neurogenic atrophy. This was thought to correlate with underlying alcohol-related or endocrine myopathy. There were no features of metabolic, mitochondrial, inflammatory or necrotising myopathies. A whole body computed tomography (CT)-^18^fluorodeoxyglucose (FDG) positron emission tomography (PET) scan identified no abnormality other than lobular increased thyroid intake, a lesion for which he had previously undergone a negative fine needle aspiration biopsy. Despite the possible spinal sensory level, magnetic resonance imaging of his spine was not undertaken given the absence of any other myelopathic features coupled with the confirmation of a severe neuropathy. Serum immunoglobulins revealed raised immunoglobulin M (IgM) at 3.65 g/L (normal range, 0.5-2.0 g/L). Electrophoresis identified no monoclonal band. Gamma-glutamyl transferase was elevated at 385 u/L (normal range, 1-71 u/L). No other cause was identified other than excessive alcohol intake prior to admission. A full blood count showed a haemoglobin level of 12.3 g/dL (normal range, 13-18 g/dL) and a mean cell volume of 104.3fL (normal range 82-98fL). Vitamin B_12 _deficiency (191 ng/L, normal range 200-900 ng/L) had been identified when our patient initially presented with symptoms, and was treated with B_12 _replacement (instituted by his general practitioner). Other relevant investigation results which were negative or did not show significant abnormalities included renal function, the remainder of liver function tests and full blood count, bone profile, coagulation screen, creatine kinase, thyroid function tests, HIV-1, HIV-2, *Borrelia burgdorferi *serology, and autoantibody testing for anti-nuclear, extractable nuclear antigen and anti-neutrophil cytoplasmic antibodies. Anti-glycolipid antibody testing revealed an elevated titre of anti-GM1 IgM antibody at 1700 units (normal range, 0-500 units). Glycated hemoglobin (HbA1C) was 5.9%.

His vitamin B1 level was measured prior to thiamine supplementation and hypovitaminosis B1 was confirmed (45 nmol/L, reference range 66-200 nmol/L), in keeping with his rapid clinical deterioration being attributable to dry beriberi.

#### Treatment

High potency intravenous (iv) multivitamins (Pabrinex) and then oral thiamine (200 mg twice a day) were commenced shortly after admission. On the basis of his anti-GM1 antibody positivity and the remaining possibility of a primary immune-mediated polyneuropathy, he received a trial of treatment with intravenous immunoglobulin. Anti-GM1 positivity was subsequently regarded to be a probable non-pathogenic epiphenomenon. Our patient also received his usual medications for established medical co-morbidities (oral hypoglycaemics and anti-hypertensives). He received gabapentin for neuropathic symptoms.

#### Progress

Within two weeks of treatment with intravenous Pabrinex and subsequent oral thiamine, our patient started to improve significantly, with dramatic improvement in limb power and reduction in sensory loss. He was discharged six weeks after admission. When subsequently followed up as an out-patient, 15-16 months from initial onset of symptoms and approximately six months after the onset of his marked tetraparesis, he was able to stand independently and was gradually gaining confidence in walking pending a period of in-patient neurorehabilitation. On examination he had grade 5 power throughout his upper limbs, and grade 4+ throughout his lower limbs. Pinprick sensation was only reduced in his legs in stocking distribution. Reflexes remained absent in his lower limbs, and were depressed in his upper limbs, although triceps jerks were easily elicited bilaterally.

## Discussion

The rates of chronic and acute alcohol abuse are increasing, with 4.6% of all ill-health and premature deaths worldwide due to alcohol [3]. For individuals who chronically consume high levels of alcohol, there is often associated liver disease which impairs storage and phosphorylation of thiamine in the liver. This inadequate liver function and low thiamine level is exaggerated by poor diet and the increased requirement for thiamine due to the continued alcohol consumption. The lack of thiamine impairs aerobic glucose metabolism which in turn causes nervous system dysfunction, given its need for glucose as an energy source [4].

The neuropathy seen in most alcoholics is usually chronic, with insidious onset, is length-dependent, and is predominantly sensory. However, the physician needs to remember that in alcoholics with dry beriberi, neuropathy can present as an acute non-length-dependent process mimicking GBS. The acute denervation changes on EMG can be profuse and widespread, mimicking motor neurone disease. The thoraco-lumbar and cranial segments are often involved and this may be deceptive to the unsuspecting mind.

Anti-GM1 antibodies have previously been found in the sera of patients with polyneuropathy and either insulin-dependent or non-insulin-dependent diabetes [5]. There are a number of possible explanations, including the independent occurrence of polyneuropathy and raised anti-GM1 antibody titre in diabetic patients, anti-GM1 antibody as an epiphenomenon associated with motor nerve damage (without direct pathogenecity), and anti-GM1 antibody mediated motor nerve destruction in some patients. Taking into account the temporal profile of our patient's clinical deterioration and the overall clinical context, it seems unlikely that anti-GM1 antibody contributed substantially to his rapid deterioration. Whilst a trial of treatment with intravenous immunoglobulin was given prior to the result of the vitamin B1 assay being available, we do not consider intravenous immunoglobulin treatment to have modified his clinical course.

It is likely that this man's background history of DM, alcohol abuse and associated nutritional deficiencies all contributed to a multifactorial initial presentation, with dry beriberi as the cause of his subsequent rapid clinical deterioration. A recent case of dry beriberi mimicking GBS [2] has been reported, although hypovitaminosis B1 had not been confirmed in that case. Although thought to be rare, we feel dry beriberi may represent an under-recognized and increasingly common cause of acute polyneuropathy, particularly against a backdrop of increasing alcohol abuse. An increasingly high index of suspicion is likely to be needed especially given that this is a treatable condition.

The clinical spectrum of beriberi encompasses dry beriberi with predominant neurological involvement and wet beriberi which mainly affects the cardiovascular system, with presentations including unexplained congestive cardiac failure [6] and lactic acidosis [7]. Bariatric surgery patients represent another group at risk of micronutrient deficiency, including B1 deficiency [8,9]. Thiamine deficiency can also occur in re-feeding syndrome [10], and in patients on parenteral nutritional support [11].

## Conclusion

A greater index of suspicion is required for nutritional hypovitaminosis B1 as a cause of rapidly worsening polyneuropathy, particularly in view of rising alcohol abuse in the western world. Confirmation of hypovitaminosis B1 by requesting the assay prior to vitamin replacement ensures accurate diagnosis and appropriate ongoing treatment.

## Consent

Written informed consent was obtained from the patient for publication of this case report. A copy of the written consent is available for review by the Editor-in-Chief of this journal.

## Competing interests

The authors declare that they have no competing interests.

## Authors' contributions

HE and AH jointly drafted the manuscript, with OK and GL contributing. GL also undertook neurophysiological examination and contributed to the discussion of differential diagnosis. All authors read and approved the manuscript.
